# Quality Criteria for Real-world Data in Pharmaceutical Research and Health Care Decision-making: Austrian Expert Consensus

**DOI:** 10.2196/34204

**Published:** 2022-06-17

**Authors:** Peter Klimek, Dejan Baltic, Martin Brunner, Alexander Degelsegger-Marquez, Gerhard Garhöfer, Ghazaleh Gouya-Lechner, Arnold Herzog, Bernd Jilma, Stefan Kähler, Veronika Mikl, Bernhard Mraz, Herwig Ostermann, Claas Röhl, Robert Scharinger, Tanja Stamm, Michael Strassnig, Christa Wirthumer-Hoche, Johannes Pleiner-Duxneuner

**Affiliations:** 1 Institute for Science of Complex Systems Center for Medical Statistics, Informatics, and Intelligent Systems Medical University of Vienna Vienna Austria; 2 Complexity Science Hub Vienna Vienna Austria; 3 Gesellschaft für Pharmazeutische Medizin Vienna Austria; 4 Medical University of Vienna Vienna Austria; 5 Gesundheit Österreich GmbH Vienna Austria; 6 Austrian Medicines and Medical Devices Agency (AGES Medizinmarktaufsicht) Vienna Austria; 7 Verband der pharmazeutischen Industrie Österreichs (PHARMIG) Vienna Austria; 8 EUPATI Austria Vienna Austria; 9 Federal Ministry of Social Affairs, Health, Care and Consumer Protection Vienna Austria; 10 Vienna Science and Technology Fund Vienna Austria

**Keywords:** real-world data, real-world evidence, data quality, data quality criteria, RWD quality recommendations, pharmaceutical research, health care decision-making, quality criteria for RWD in health care, Gesellschaft für Pharmazeutische Medizin, GPMed

## Abstract

Real-world data (RWD) collected in routine health care processes and transformed to real-world evidence have become increasingly interesting within the research and medical communities to enhance medical research and support regulatory decision-making. Despite numerous European initiatives, there is still no cross-border consensus or guideline determining which qualities RWD must meet in order to be acceptable for decision-making within regulatory or routine clinical decision support. In the absence of guidelines defining the quality standards for RWD, an overview and first recommendations for quality criteria for RWD in pharmaceutical research and health care decision-making is needed in Austria. An Austrian multistakeholder expert group led by Gesellschaft für Pharmazeutische Medizin (Austrian Society for Pharmaceutical Medicine) met regularly; reviewed and discussed guidelines, frameworks, use cases, or viewpoints; and agreed unanimously on a set of quality criteria for RWD. This consensus statement was derived from the quality criteria for RWD to be used more effectively for medical research purposes beyond the registry-based studies discussed in the European Medicines Agency guideline for registry-based studies. This paper summarizes the recommendations for the quality criteria of RWD, which represents a minimum set of requirements. In order to future-proof registry-based studies, RWD should follow high-quality standards and be subjected to the quality assurance measures needed to underpin data quality. Furthermore, specific RWD quality aspects for individual use cases (eg, medical or pharmacoeconomic research), market authorization processes, or postmarket authorization phases have yet to be elaborated.

## Introduction

Real-world data (RWD) is an overarching term for data on patient’s health (health status, effectiveness, medical treatment, the pattern of use of medicinal products, and resource use, etc) that are collected in routine health care processes and not in the context of clinical trials. RWD involve large and complex data sets such as data from electronic health records, pharmacy data, electronic smart devices, patient-reported outcomes, and digital applications or platforms [1,2]. When RWD are analyzed, they lead to real-world evidence (RWE) on the pattern of use and effectiveness of any kind of procedure, drug, or nonpharmacological intervention. The availability of RWD and evolving analytic techniques to generate RWE have created interest within the research and medical communities to use RWD and RWE to enhance clinical research and support regulatory decision-making [1,3]. On a European level, the European Medicines Agency (EMA) and Heads of Medicines Agencies fully recognize the value of health data and set up a joint task force to describe the health data landscape from a regulatory perspective and identify practical steps for the European medicines regulatory network to make the best use of health data in support of innovation and public health in the European Union [4].

The comprehensive work plan identifies 10 priorities [5], such as delivering a sustainable platform to access and analyze health care data from across the European Union (Data Analysis and Real World Interrogation Network [6]) or establishing an EU framework for data quality (European Health Data & Evidence Network [7] and Health Outcomes Observatory [8]) and representativeness. Despite many initiatives, there are still no guidelines for the quality criteria that RWD must meet in order to be able to use it for decision-making purposes within regulatory or routine clinical decision support. As a first example, the EMA Guideline on registry-based studies [9] provides considerations on good practice for registries to increase their usefulness for regulatory purposes.

The objective of this consensus statement of the Austrian Expert Group led by Gesellschaft für Pharmazeutische Medizin (GPMed; Austrian Society for Pharmaceutical Medicine) is to provide an overview and first recommendations for the quality criteria of RWD for primary and secondary research purposes to be adopted in medical or pharmacoeconomic research and health care decision-making processes. The consensus statement does not discuss the general use of RWD nor how to obtain RWE in general.

## Methods

After EMA published a drafted guideline for registry-based studies, interested GPMed board members volunteered together with Austrian Medicines and Medical Devices Agency executive experts to assess how ready the Austrian research landscape is for registry-based studies.

The Austrian Medicines and Medical Devices Agency and GPMed invited Austrian RWD researchers and data experts to contribute voluntarily to the topic. The criteria to select working group members were those with scientific work in the field and longstanding expertise in using RWD for research purposes. After the kickoff meeting in April 2021, the expert group led by GPMed met on a monthly basis; reviewed guidelines, frameworks, use cases, or viewpoints; and derived a consensus statement on the quality criteria for RWD to be used more effectively for medical research purposes beyond the registry-based studies discussed in the EMA Guideline for registry-based studies [9].

Following agreement on a joint definition on RWD, experts from the group shared examples of RWD frameworks, guidelines, or viewpoints, which were discussed in the working group, and consensus was reached unanimously within the monthly meetings.

## Results

### Definition of RWD

Despite an increasing recognition of the value of RWD, a global consensus on the definition of RWD is lacking [10]. The definition of RWD can differ in various areas of application (eg, public health vs automotive industry). However, the expert group led by GPMed reviewed several definitions [7,8,10-15] and agreed on the following description.

Real-world data can be defined as data relating to patient health status or the delivery of health care that are routinely collected from a variety of sources (including patient-reported outcomes), such as:

- health care databases (systems into which health care providers routinely enter clinical and laboratory data; eg, electronic health records and pharmacist databases),
- health insurance and claims databases (maintained by payers for reimbursement purposes),
- patient registries (data on a group of patients with specific characteristics in common),
- disease registries (data on a particular disease or disease-related patient characteristic regardless of exposure to any medicinal product, other treatment, or a particular health service),
- data gathered from other sources that can inform on health status, such as mobile devices, wearables, or other smart medicinal products (eg, real-time continuous glucose monitoring devices),
- social media– and patient-powered research networks (eg, patient networks to share health information),
- biobanks, and
- observational studies.

Note that this definition includes data that are neither collected by licensed medical devices operated by health professionals in clinical settings nor observational data that are typically stored in public health registries and administrative databases. Namely, RWD also include health-related data that are generated by the patient by means of digital health technologies (sensors, wearables, and smartphones, etc). Hence, ethical and regulatory frameworks should also be applied to these health-related data and not only target health care databases and registries [16].

### Examples of RWD Frameworks

Globally and Europe-wide, more and more examples of how RWD are used for research or regulatory purposes are being published. The expert group decided to illustrate some examples of how the quality of RWD is ensured along different approaches (Table 1). Further details to this overview can be found in the Multimedia Appendix 1.

**Table 1:** Examples and short descriptions of reviewed real-world data (RWD) frameworks.

RWD framework	Short description	Country
RWD for health systems research [17-23]	Nordic countries have set the worldwide gold standard for how RWD can be leveraged. Good RWD frameworks exist in Finland, Denmark, Sweden, Iceland, and Norway. The RWD quality and infrastructure built up in these countries can be seen as best practice examples for how to leverage RWD for research.	Denmark, Finland, Iceland, Norway, and Sweden
Danish Data Analytics Center [24]	The Danish DAC^a^ has access to some of the most sophisticated and complete patient-level health data in the world and meets the highest requirements for data and IT security. DAC constitutes a unique possibility for the use of big data analytics to discover hidden patterns to benefit patients. It will reduce the entry barriers for new drugs to go to market while maintaining the high safety standards currently in place.	Denmark
EMA^b^ submission supported by historical cohort patient data [25]	Based on the observed efficacy in Phase 2 studies (n=189 and n=36) and combined with an additional historical comparator study (1139 cases), conditional marketing authorization was granted with the need to better quantify the magnitude of the effect by submitting data from a Post Authorization Efficacy Study (Phase 3 randomized, comparative study of blinatumomab vs standard of care chemotherapy) as well as a noninterventional Post Authorization Safety Study in subsequent years.	European Union
Demonstrated the research potential of a clinico-genomic database [26,27]	In 2017, Foundation Medicine and Flatiron Health created a proof-of-concept study. Using a sample size of over 2000 patients with non–small cell lung cancer, they discovered that high versus low tumor mutation burden showed a far stronger association than high versus low PD-L1 levels after immunotherapy. Their results were nearly identical to those derived by a drug manufacturer from a post hoc analysis of a failed clinical trial. The validation study helped establish the groundwork for this data set to be used to advance cancer research.	United States
Multidatabase studies for medicines surveillance in real-world settings [28,29]	Postmarketing studies can be underpowered if outcomes or exposure of interest are rare, or the interest is in the subgroup effects. Combining several databases might provide the statistical power needed. Although many multidatabase studies have been performed in Europe in the past 10 years, there is a lack of clarity on the peculiarities and implications of the existing strategies to conduct them. Experts identified 4 strategies to execute multidatabase studies, classified according to specific choices in the execution.	European Union
EUnetHTA^c^ REQueST^d^ [30]	The Registry Evaluation and Quality Standards Tool (REQueST) aims to support health technology assessment organizations and other actors in guiding and evaluating registries for effective use in health technology assessment.	European Union

^a^DAC: Data Analytics Center.

^b^EMA: European Medicines Agency.

^c^EUnetHTA: European Network for Health Technology Assessment.

^d^REQueST: Registry Evaluation and Quality Standards Tool.

### Legal Frameworks

The current legal framework in Austria with the Federal Statistics Act as well as the Research Organization Act recognizes the “use” of RWD—especially for research purposes [31-33].

Independently of the question of data availability, many RWD sources, as defined within this expert consensus paper, do not address data quality issues. Therefore, the need for high–data quality standards should be also recognized by legal frameworks. On a European level, data quality aspects are strongly embedded within the development of the European Health Data Space [34] and Data Analysis and Real World Interrogation Network [6]. Shared outcomes on data quality should be reflected within local legal frameworks as well.

### Recommendations

#### Data Quality

RWD are often used for purposes that are different from the intention for which the data were collected originally. Therefore, it is of utmost importance to check upfront if the RWD are adequate in terms of clearly defined quality criteria and can, therefore, be used in general for primary or secondary research purposes as well. Due to the lack of guidelines defining the quality standards of RWD to be used for decision-making, it is even more important to be able to assess the suitability of RWD for research purposes by applying checklists and some standardized questionnaires [35-38].

#### RWD Should Follow High Standards and Be Subject to Quality Assurance

The value of the secondary use of RWD data (in particular, registries) for research purposes depends crucially on their quality as quantified by *completeness* and *accuracy* [39], next to *timeliness*, *comparability*, the technical prerequisite that the size of the data source is sufficient (ie, the study does not become underpowered), and that the data is in principle accessible and can be mapped with other relevant data sets (well defined research question outlined in a research plan). An evaluation with regard to these factors is therefore recommended before using the data. Note that these quality criteria are not unique in the sense that alternative data quality concepts have also been described (eg, validity, consistency, and integrity).

*Completeness* is defined as the proportion of true cases of a variable (disease, treatment, and diagnose, etc) in all or a certain subgroup of patients that is correctly reported in the data. Completeness therefore captures the amount of missing data in a specific source—the extent to which all necessary data that could have been registered has been registered [40]. Very often there is no comprehensive reference source available for evaluating the completeness of a data set with regard to the general population. In that case, it might be advisable to identify studies that report the variables of interest for specific comparable subgroups and therefore allow for an assessment of data completeness [39]. These comparisons should ideally be performed on an individual level (eg, comparing data records from registries for certain diseases to administrative records) or, in cases where the required information is not available on an individual level, attempts should be made to examine completeness at least on an aggregate level (by comparing the expected number of cases across data sets).

*Accuracy* measures the proportion of patients with a certain property (diagnosis, prescription, and socioeconomic or demographic properties, etc) in a data set that truly have the property. Accuracy is typically assessed by comparing the data records with the reference standard used to confirm the specific variable [41]. In many cases, this reference could be the medical record; for certain areas, other references might be feasible as well. One strategy to perform such a comparison could be to randomly sample a given percentage (eg, 5%) or an absolute number (eg, 1000) manually. This helps to identify errors and whether they are systematic (as often happens through algorithmic problems when the data are collected in an automated way or if the data are collated from different reporting systems, regional or otherwise) or random (often resulting from manual data collection), thereby informing strategies to increase data accuracy.

*Timeliness* measures data quality with regard to the time at which the variable (disease and diagnosis, etc) was recorded (eg, the extent to which the time of the recorded disease corresponds to the true time of the disease). This can often be assessed together with completeness and accuracy and is of particular importance in longitudinal study designs.

Furthermore, *comparability* needs to be checked to ensure that variable definitions in a data set conform to international guidelines and other relevant references.

A comprehensive review of 114 data quality studies in the Danish registry network showed that both completeness and accuracy increased over time and accuracy varies substantially across different diseases, between less than 15% of correctly coded diagnoses to almost 100% [41]. This finding underscores the *need for data quality assurance of RWD for research use*.

#### High Research Standards Should Underpin the Quality of RWD

##### Study Protocol

Observational postmarketing studies are an important tool, using data obtained from routine clinical care, to provide data on medical treatment effect estimates and the tolerability of medicinal products in a real-world setting, as well as for medical devices as part of the postmarketing surveillance [42]. Nonrandomized studies may be used to complement the evidence base represented by randomized controlled trials [43], even though one cannot expect nonrandomized, observational studies to exactly reproduce randomized controlled trials as these are different study designs, and hence measure different types of effects [44]. Noncontrolled studies lack a comparison group, which means that inferences on the treatment effect and tolerability must rely on before-and-after comparisons of the outcome of interest. Treatment effect estimates and tolerability derived from nonrandomized studies are at greater risk of bias. Thus, data from routine clinical observation should be collected after the development of a study protocol where the population of interest, study outcome, methods for data generation and analysis, limitation of study data, and bias are defined in advance, as also defined in the EMA guideline for registry-based studies [9].

##### Informed Consent

The informed consent process of patients in observational, noninterventional studies are not discussed by Good Clinical Practice (ISO 14155) [45], and this topic is still dealt with heterogeneously throughout the European Union. Within the study protocol, the consent process and requirements of compliance to the General Data Protection Regulation (GDPR) should be specified. Data generated in an anonymized way would not require patient consent, though collection of pseudonymized data in observational studies requires the consent of patients prior to data collection, which should be limited only to the GDPR requirements, and not include any consent to medical treatment. The burden of obtaining informed consent to collect routine clinical data should be kept feasible to reduce bias of missing data from severely ill patients or patients incapable of consenting, such as in emergency situations. Since GDPR applies only to living people, a waiver for data collection from the deceased can be obtained if the purpose is sufficiently outlined in the study protocol.

##### Institutional Review Board and Ethics Committee

Within the study protocol, all interventions in the observational trial (ie, treatment, diagnostic or monitoring procedures) should fall within the standard of care or routine treatment, as interpreted by the competent authority or ethics committee in that member state. Thus, a review and approval from the respective ethics committee is required, as also indicated in the EMA guideline for registry-based studies [9].

### Checklist on Quality Criteria for RWD

Following general recommendations and reflecting guidelines and checklists on registry-based research [9,37], the expert group suggests a minimum set of criteria summarized within the checklist presented in Table 2 to ensure the quality of RWD for research purposes and health care decision-making processes.

**Table 2:** Gesellschaft für Pharmazeutische Medizin (GPMed) checklist for real-world data (RWD) quality.

Criteria	Description
Data management and stewardship	“FAIR Data Principles” which formulate principles that sustainable, reusable research data and research data infrastructures must meet [38,46,47]
Governance framework	Available policy for collaborations with external organizations Involvement of patient organizations Governance structure for decision-making on requests for collaboration Templates for research and data-sharing contracts between partners and institutions
Quality requirements	High–RWD quality standards are implemented, such as completeness, accuracy, timeliness, and comparability Process in place for ongoing data quality assessments Processes in place for quality planning, control, assurance, and improvement Data verification (the method and frequency of verification) Auditing practice
Data privacy and transparency	Informed consent processes and its validity for research purposes according to General Data Protection Regulation and relevant national regulations Data privacy officer
Research objectives	Well-defined research question outlined in a research plan Available documentation, protocol, or proposal that describes the purpose of RWD use and rational that the RWD sources adequately address the research questions (eg, study protocol) Approval of RWD use from independent an institutional review board or ethics committee Protocol should follow the Declaration of Helsinki, and furthermore, the Declaration of Taipei [48] on Research on Health Databases, Big Data and Biobanks should be taken into account
Data providers	Adequate description of data providers, such as patients, caregivers, or health care professionals; their geographical area; and any selection process (inclusion and exclusion criteria) that may be applied for their acceptance as data providers
Patient population covered	Adequate description of the type of patient population (disease, condition, time period covered, and procedure), which defines the criteria for patient eligibility Relevance of setting and catchment area Clarity on patients’ inclusion and exclusion criteria Methods applied to minimize selection bias and loss to follow-up Ensure fair representations of minorities, sex, gender, and socially disadvantaged groups
Data elements	Core RWD set collected for RWD use case or purpose Definition, dictionary, and format of data elements Standards and terminologies applied Capabilities and plans for amendments of data elements
Infrastructure	High-quality systems for RWD collection, recording, and reporting, including timelines Capability (and experience) for expedited reporting and evaluation of severe suspected adverse reactions in RWD collection Capability (and experience) for periodic reporting of clinical outcomes—ideally patient-reported outcomes—and adverse events reported by physicians, at the individual-patient level and aggregated data level Capability (and experience) for data cleaning, extraction, transformation, and analysis Capability (and experience) for data transfer to external organizations Capabilities for amendment of safety reporting processes

## Discussion

### Principle Findings

Over the past months, EU and EMA strategies, workplans, and initiatives on health data use developed very quickly [34,49-51]. This paper shows the consensus of a multistakeholder expert group which summarizes a minimum set of the quality criteria of RWD for research and decision-making purposes in health care. The most important quality assurance measures identified are a profound data management and stewardship; established governance framework; standardized quality requirements; adhered data privacy and transparency measures; well-defined research objectives; adequate description of data providers; well-described patient population covered; outlined which data elements are required; and high-quality infrastructure for RWD collection, recording, and reporting.

### Conclusions

To future-proof registry-based studies, the group strongly recommends that RWD should follow high standards and be subject to the quality assurance measures needed to underpin the quality of RWD. Furthermore, specific RWD quality aspects for individual use cases (eg, medical or pharmacoeconomic research), market authorization processes, or postmarket authorization phases have yet to be elaborated.

## Appendix

Multimedia Appendix 1 Examples of real-world data frameworks or use cases.

## Abbreviations

EMA: European Medicines Agency
GDPR: General Data Protection Regulation
GPMed: Gesellschaft für Pharmazeutische Medizin
RWD: real-world data
RWE: real-world evidence
