# Supplementary material for: Quality Criteria for Real-world Data in Pharmaceutical Research and Health Care Decision-making: Austrian Expert Consensus
Source: JMIR Med Inform. 2022 Jun 17;10(6):e34204. doi: 10.2196/34204 (PMC9250059; doi:10.2196/34204)
Supplement: Multimedia Appendix 1 [file medinform_v10i6e34204_app1.docx]

**Multimedia Appendix to “Quality criteria for Real-World Data in pharmaceutical research and healthcare decision making. An Austrian Expert Consensus.”**

Klimek P, Baltic D, Brunner M, Degelsegger-Marquez A, Garhöfer G, Gouya-Lechner G, Herzog A, Jilma B, Kähler S, Mikl V, Mraz B, Ostermann H, Röhl C, Scharinger R, Stamm T, Strassnig M, Wirthumer-Hoche C, Pleiner-Duxneuner J.

## Multimedia Appendix 1: Examples of RWD frameworks or use cases

### RWD for Health Systems Research

Nordic countries have set the world-wide gold standard for how RWD can be leveraged for health systems research through the establishment of extensive networks of linkable longitudinal population-wide registries [27].

Typically, these networks are made up of administrative medical claims datasets (outpatient, inpatient and emergency department contacts) that can be linked at the patient level with data from other administrative (e.g., birth, death) or clinical registers, next to data from randomized controlled trials, populations surveys, or epidemiological field studies.

Such linked RWD for health systems research and planning have existed in Finland since 1969 [28], in Denmark since 1978 [29], in Sweden since 1987 [30], in Iceland since 1999 [31] and in Norway since 2008 [32]. The data infrastructures built up in these countries can be seen as best practice examples for how to leverage RWD for research. Use cases for such data have been summarized extensively in Schmidt et al. [33].

Next to risk studies for the occurrence of disease in the general population in relation to certain exposures, such RWD can be used for cohort studies (hospitalized population, family cohorts, etc.), case-control studies (through identification of cases and suitable controls from the registers), cross-sectional studies (analysing patient variables and history at study entry time which is given by certain index events) and ecological studies (variations in health-related variables at population level). Through linkage of datasets at the level of individual patients it becomes possible to identify cohorts, their medical history and outcome variables, as well as diseases, treatments, diagnostic examinations or other health-related variables as exposures across multiple individual datasets.

### Danish Data Analytics Center (DAC) [34]

Denmark has one of the most extensive health data registries in the world. The large amount of patient-level data on every aspect from treatment regimens over reported adverse drug reactions to socioeconomic factors constitutes a unique possibility for the use of big data analytics to discover hidden patterns to the benefit of the patients.

Another aspect of the use of data analytics on health data is the potential to create a new paradigm for approval of new medicines. By tracking adverse drug reactions on a large scale in real time, it will be possible for the regulators to approve drugs faster and act on safety signals, if unexpected adverse drug reactions present themselves. It will reduce the entry barriers for new drugs to go to market while maintaining the high safety standards currently in place.

- DAC is the Data Analytics Centre of the Danish Medicines Agency located in Copenhagen, Denmark.
- DAC will work to transform information and data on medicine and medicine devices into knowledge that can benefit the citizens.
- DAC will enable the Danish Medicines Agency to offer new types of scientific advice to the pharmaceutical companies on the development of new medicines, including medicines for people with rare diseases. DAC will also develop new methods for optimized real time drug safety surveillance.
- DAC uses a wide range of different data sources. Via the Danish Health Data Authority, The Danish Clinical Quality Program – National Clinical Registries and others, DAC has access to some of the most sophisticated and complete patient-level health data in the world. DAC analyses are run on Danish National Genome Centre supercomputer, which meets the highest requirements for data and IT-security.

### EMA submission supported by historical cohort patient data [36]

The application for Marketing Authorization of Blinatumomab for the treatment of adults with Philadelphia chromosome negative relapsed or refractory B-precursor acute lymphoblastic leukaemia (ALL) has been submitted to EMA in 2014.

Due to the orphan nature of this specific subtype of ALL (incidence in the EU appr. 900 patients/year), the submission has been based on two small Phase-2 open-label, uncontrolled, single-arm clinical studies (n=189 and n=36) but supported by a historical comparator study and a model-based literature meta-analysis. The historical comparator study provided subject level historical data on hematological remission rates and survival in this specific patient population treated with standard of care chemotherapy and included data from 1,139 patients whose initial ALL diagnosis was after 01.01.1990.

Based on the observed efficacy in Phase-2 studies and combined with these additional analyses, conditional marketing authorization granted with the need to better quantify the magnitude of the effect by submitting data from a PAES (Phase-3 randomized, comparative study of blinatumomab versus standard of care chemotherapy) as well as a non-interventional PASS in subsequent years.

### Demonstrated research potential of clinico-genomic database [37,38]

In 2014, Foundation Medicine and Flatiron partnered on an ambitious project to combine a subset of their data into the first-of-its-kind clinico-genomic database (CGDB).

In early 2017, to demonstrate the CGDB’s research potential, Foundation Medicine and Flatiron created a proof-of-concept study. Specifically, they examined how lung cancer patients responded to an approved immunotherapy treatment based on two biomarkers: PD-L1 and Tumour Mutational Burden (TMB). Using a sample size of just over 2,000 patients with non-small cell lung cancer, they discovered that high versus low TMB has a far greater impact than high versus low PD-L1 on response to immunotherapy.

Their results were nearly identical to those derived by a drug manufacturer from a post-hoc analysis of a failed clinical trial. The striking difference was that using the CGDB, the researchers completed their work in a matter of weeks at a relatively low cost. The researchers conducted a systematic comparison of analyses of the CGDB with over a dozen seminal findings in the molecular treatment of lung cancer discovered through traditional approaches. They found that they were able to recapitulate each and every one of these findings.

This validation study [38] helps establish the necessary groundwork for this dataset to be used to advance cancer research.

### Multi-Database Studies for Medicines Surveillance in Real-World Setting [39,40]

Although post-marketing studies conducted in population-based databases often contain information on patients in the order of millions, they can still be underpowered if outcomes or exposure of interest is rare, or the interest is in subgroup effects. Combining several databases might provide the statistical power needed.

A multi-database study (MDS) uses at least two healthcare databases, which are not linked with each other at an individual person level, with analyses carried out in parallel across each database applying a common study protocol. Although many MDSs have been performed in Europe in the past 10 years, there is a lack of clarity on the peculiarities and implications of the existing strategies to conduct them. In this review, the authors identify four strategies to execute MDSs, classified according to specific choices in the execution [39]:

- local analyses, where data are extracted and analysed locally, with programs developed by each site;
- sharing of raw data, where raw data are locally extracted and transferred without analysis to a central partner, where all the data are pooled and analysed;
- use of a common data model with study-specific data, where study-specific data are locally extracted, loaded into a common data model, and processed locally with centrally developed programs; and
- use of general common data model, where all local data are extracted and loaded into a common data model, prior to and independent of any study protocol, and protocols are incorporated in centrally developed programs that run locally.

### EUNetHTA REQueST Tool [41]

The Registry Evaluation and Quality Standards Tool (REQueST) aims to support HTA organisations and other actors in guiding and evaluating registries for effective usage in HTA.

The tool has been developed to be a comprehensive resource that covers all important aspects relating to the quality of registries. The standards set out in the tool are universal and essential elements of good practice and evidence quality that are, therefore, relevant for different types of registries. REQueST sits alongside the existing guidance on registries as a way to implement it.

REQueST is designed to be used by i) evidence developers to assess the quality of their registry, and ii) international organisations considering whether to use registry data for HTA and regulatory purposes. The purpose is to highlight areas of a registry that need improvement in order to maximise the quality of its data and ensure that those data can be used for HTA and regulatory purposes.

## References

1. Sørensen HT. Regional administrative health registries as a resource in clinical epidemiology: a study of options, strengths, limitations and data quality provided with examples of use. Int J Risk Saf Med. 1997;10(1):1–22.
2. Sund R. Quality of the Finnish hospital discharge register: a systematic review. Scand J Public Health. 2012;40(6):505–515.
3. Lynge E, Sandegaard JL, Rebolj M. The Danish national patient register. Scand J Public Health. 2011;39(7 Suppl):30–33.
4. Ludvigsson JF, Andersson E, Ekbom A, et al. External review and validation of the Swedish national inpatient register. BMC Public Health. 2011;11(1):450.
5. Gudbjornsson B, Thorsteinsson SB, Sigvaldason H, et al. Rofecoxib, but not celecoxib, increases the risk of thromboembolic cardiovascular events in young adults – a nationwide registry-based study. Eur J Clin Pharmacol. 2010;66(6):619–625.
6. Helsedirektoratet Norsk pasientregister. Available from: <http://www.helsedirektoratet.no/kvalitet-planlegging/norsk-pasientregisternpr/innhold-og-kvalitet/Sider/default.aspx>. [accessed Sep 21, 2021]
7. Schmidt M, Schmidt SAJ, Sandegaard JL, Ehrenstein V, Pedersen L and Sorensen HT. The Danish National Patient Registry: a review of content, data quality, and research potential. Clin Epidemiol. 2017; 7:449-490.
8. Data Analytics Centre of the Danish Medicines Agency. Copenhagen. <https://laegemiddelstyrelsen.dk/en/about/organisation/name/> [accessed Sep 21, 2021]
9. Regulation (EU) 2017/745 of the European Parliament and of the Council 2017 on medical devices, amending Directive 2001/83/EC, Regulation (EC) No 178/2002 and Regulation (EC) No 1223/2009 and repealing Council Directives 90/385/EEC and 93/42/EEC
10. EMA/CHMP/469312/2015. Assessment report blinatumomab. Procedure No. EMEA/H/C/003731/0000
11. Roche. Getting closer to cancer research's holy grail: The Clinico-Genomic Database. <https://www.roche.com/about/priorities/personalised_healthcare/combining-data-to-advance-personalised-healthcare.htm> [accessed May 21, 2021]
12. Singal G, Miller PG, Agarwala V, et al. Association of Patient Characteristics and Tumor Genomics With Clinical Outcomes Among Patients With Non–Small Cell Lung Cancer Using a Clinicogenomic Database. JAMA. 2019;321(14):1391–1399. doi:10.1001/jama.2019.3241
13. Gini R, Sturkenboom MCJ et al; Working Group 3 of ENCePP (Inventory of EU data sources and methodological approaches for multisource studies). Different Strategies to Execute Multi-Database Studies for Medicines Surveillance in Real-World Setting: A Reflection on the European Model. Clin Pharmacol Ther. 2020 Aug;108(2):228-235. doi: 10.1002/cpt.1833. Epub 2020 May 5. PMID: 32243569; PMCID: PMC7484985.
14. Real world research on medicines: European Network of Centres in Pharmacoepidemiology and Pharmacovigilance (ENCePP), <https://www.ema.europa.eu/en/events/real-world-research-medicines-contribution-european-network-centres-pharmacoepidemiology#event-summary-section> [accessed May 21, 2021]
15. EUNetHTA ReQUEst Tool. <https://eunethta.eu/request-tool-and-its-vision-paper/> [accessed May 21, 2021]
16. European Health Data Space. <https://ec.europa.eu/health/ehealth/dataspace_en> [accessed May 4, 2022]
17. TEHDAS. Towards European Health Data Space. <https://tehdas.eu/> [accessed May 4, 2022]
18. Big Data Steering Group Workplan 2021-2023. <https://www.ema.europa.eu/en/documents/work-programme/workplan-2021-2023-hma/ema-joint-big-data-steering-group_en.pdf> [accessed May 4, 2022]
19. Electronic cross-border health services. <https://ec.europa.eu/health/ehealth/electronic_crossborder_healthservices_en> [accessed May 4, 2022]
